# Usability of a Web-Based App for Increasing Adolescent Vaccination in Primary Care Settings: Think-Aloud and Survey Assessment

**DOI:** 10.2196/56559

**Published:** 2024-09-19

**Authors:** Stephanie A S Staras, Justin Tauscher, Michelle Vinson, Lindsay A Thompson, Mary A Gerend, Elizabeth A Shenkman

**Affiliations:** 1 Department of Health Outcomes and Biomedical Informatics College of Medicine University of Florida Gainesville, FL United States; 2 Behavioral Research in Technology and Engineering (BRiTE) Center Department of Psychiatry and Behavioral Sciences University of Washington Seattle, WA United States; 3 Network for Clinical Research and Training College of Medicine Florida State University Orlando, FL United States; 4 Department of Pediatrics Wake Forest University Winston-Salem, NC United States; 5 Department of Behavioral Sciences and Social Medicine College of Medicine Florida State University Tallahassee, FL United States

**Keywords:** participatory design, think-aloud, implementation science, adolescent vaccination, human papillomavirus vaccine, usability, eHealth

## Abstract

**Background:**

In the United States, only 58% of teens receive the recommended 2 doses of the human papillomavirus vaccine by 15 years of age. Overcoming vaccine hesitancy often requires effective communication between clinicians and parents to address specific concerns. To support this, we developed ProtectMe4, a multilevel, theory-informed web-based intervention designed to address parents’ vaccine-related questions and assist clinicians in discussing vaccine concerns for 4 adolescent vaccines.

**Objective:**

This study aims to evaluate the usability of ProtectMe4 in routine care settings across 3 pediatric primary care clinics. Specifically, the study aims to (1) observe the proposed workflow in practice, (2) identify usability issues experienced by parents and clinicians, and (3) assess the perceptions of both parents and clinicians regarding the app’s usability.

**Methods:**

On designated days in 2020 and 2021, the study team recruited parents of 11- to 12-year-old patients attending appointments with participating clinicians. We conducted think-aloud assessments during routine care visits and administered a usability survey after participants used the app. For parents, we simultaneously video-recorded the app screens and audio-recorded their commentary. For clinicians, observational notes were taken regarding their actions and comments. Timings recorded within the app provided data on the length of use. We reviewed the recordings and notes to compile a list of identified issues and calculated the frequencies of survey responses.

**Results:**

Out of 12 parents invited to use the app, 9 (75%) participated. Two parents who were invited outside of the planned workflow, after seeing the clinician, refused to participate. For the parents whose child’s vaccination record was identified by the app, the median time spent using the app was 9 (range 6-28) minutes. Think-aloud assessment results for parents were categorized into 2 themes: (1) troubleshooting vaccine record identification and (2) clarifying the app content and purpose. Among the 8 parents who completed the survey, at least 75% (6/8) agreed with each acceptability measure related to user satisfaction, perceived usefulness, and acceptance. These parents’ children were patients of 4 of the 7 participating clinicians. Consistent with the planned workflow, clinicians viewed the app before seeing the patient in 4 of 9 (44%) instances. The median time spent on the app per patient was 95 (range 5-240) seconds. Think-aloud assessment results for clinicians were grouped into 2 themes: (1) trust of app vaccine results and (2) clarifying the app content. On the survey, clinicians were unanimously positive about the app, with an average System Usability Scale score of 87.5 (SE 2.5).

**Conclusions:**

This mixed methods evaluation demonstrated that ProtectMe4 was usable and acceptable to both parents and clinicians in real-world pediatric primary care. Improved coordination among clinic staff is needed to ensure the app is consistently offered to patients and reviewed by clinicians before seeing the patient.

## Introduction

More effective, accurate, and tailored communication is needed between clinicians and patients or caregivers to improve the quality of health care. One area requiring improvement is the reduction of vaccine hesitancy—delay in acceptance or refusal of vaccines despite their availability—which has been identified as one of the top 10 global health threats by the World Health Organization [1]. Poor communication can undermine vaccine acceptance, while effective communication can positively influence the key determinants: confidence (trust in vaccine safety and effectiveness), complacency (not perceiving the disease as high risk or the vaccine as important), and convenience (practical barriers) [2]. In the United States, for most vaccines, including the human papillomavirus (HPV) vaccine, this communication typically takes place in primary care clinics between the clinician and the patient or parent.

Various interventions aimed at improving clinician recommendations for the HPV vaccine—primarily electronic health record reminders and communication trainings—show a pooled increase in HPV vaccine initiation (receipt of the first dose) of 13% (risk ratio 1.13, 95% CI 1.07-1.19) [3]. Interventions targeting parent vaccine acceptance, such as providing educational materials through digital and nondigital platforms and sending reminder messages, show a pooled increase in adolescent vaccination of 19% (risk ratio 1.19, 95% CI 1.12-1.26) [3]. The most effective interventions target multiple levels, such as both clinicians and parents. More tools are needed to promote HPV vaccination in the United States, as only 58% of teens meet the national health target of receiving the 2 recommended doses by the age of 15 years [4,5].

A variety of digital health interventions aim to improve the quality of conversations between clinicians and parents regarding the HPV vaccine [6]. Digital health interventions targeting clinicians focus on providing education about the HPV vaccine and strategies for how clinicians should present their recommendations and address questions [7-9]. These tools are designed primarily as general continuing education and typically lack interactive features or patient-specific tailoring advice. Digital health interventions aimed at parents and adolescents mainly offer HPV vaccine education outside of the clinical care setting [10-13]. While these apps were well received by parents and adolescents and led to increased HPV vaccine knowledge, they produced only minimal changes in HPV vaccination rates [6]. By contrast, videos that address parents’ specific concerns have been effective in increasing parents’ intent to vaccinate their child [14]. Moreover, while studies consistently show that clinicians feel unprepared to address parents’ HPV vaccine questions and recognize that clinicians are a critical source of vaccine information [15-18], we are unaware of any intervention that offers clinicians’ tailored responses to parent questions in real-time.

To address this gap, we developed *ProtectMe4*, a multilevel, theory-informed web-based intervention designed to assist with answering parents’ questions about vaccines and support clinicians in discussing specific vaccine concerns. At the parent level, the app provides a list of patient-specific recommended adolescent vaccinations—HPV; meningococcal (meningococcal conjugate vaccine against serogroups A, C, W, and Y [MenACWY]); tetanus, diphtheria, and pertussis (Tdap); and influenza—based on real-time data from the state immunization registry. It also allows parents to indicate vaccine interest, report hesitations, and access tailored educational information [19]. The Florida state immunization registry is updated in real-time with data from participating clinics across the state, providing records of all vaccines administered by health departments and participating clinicians. It contains 2 or more vaccine records for 74% of Florida’s 11- to 17-year-olds [20]. At the clinician level, the app displays patients’ vaccine records, parents’ responses regarding vaccine interest and hesitations, and offers tips on how to address specific hesitations. The main objective is to assist clinicians in conducting tailored conversations and to enhance their self-efficacy by providing just-in-time education. In line with World Health Organization recommendations for tailored vaccine education, ProtectMe4 delivers customized information to parents directly through the app and immediately before the clinician’s conversation [21].

ProtectMe4 is an updated version of an app we previously pilot tested called Protect Me from HPV, which was one of the most effective multilevel interventions for increasing HPV vaccination [3]. The app increased HPV vaccine initiation among 11- to 12-year-olds by 50% (risk ratio 1.5, 95% CI 1.2-1.9) [22]. However, because only 8% of families randomly assigned to the app were invited to use it, we applied a participatory design strategy to enhance the app’s implementation and potentially increase its effectiveness. Guided by the Diffusion of Innovation Theory [23], a component of the Consolidated Framework for Implementation Research [24], we aimed to enhance the app’s efficiency (targeting complexity), user benefits (targeting relative advantage), and workflow integration (targeting compatibility) through a series of studies. Initially, we expanded our HPV vaccine–focused app to include the 3 additional adolescent vaccines. Clinicians in our pilot study reported that the app had limited benefits because they still needed to review records for these other vaccines. Additionally, the prevailing HPV vaccine recommendation strategy suggests normalizing HPV vaccination by recommending all adolescent vaccines together [25]. Second, we redesigned the app interface to reduce complexity and integrated feedback from parents and clinicians collected through focus groups [26]. Third, we conducted a time-series workflow assessment at 4 pediatric clinic practices to determine the best way to integrate the app into real-world settings [27].

In this paper, we present the next step in the participatory design process: evaluating usability—how effectively users can achieve the app’s goals with ease and obtain what they need without encountering errors [28,29]. Usability is a critical determinant of implementation, as apps that are easier to use are more likely to be adopted and used over time [30,31]. To gather clinician and parent perspectives on the app’s usability, our study had 3 main objectives. First, we aimed to observe how a slightly altered version of the proposed workflow performed in real-world clinical settings. Second, we sought to identify usability issues with the app’s content and flow through a think-aloud test [32]. Third, we aimed to evaluate clinicians’ and parents’ perspectives on factors that might affect the app’s implementation, including acceptability (whether the app is satisfactory), appropriateness (whether the app is relevant), and feasibility (whether the app can be used effectively in a clinical setting) [33]. The purpose of this evaluation is to lay the groundwork for a feasibility test to determine whether the implementation of ProtectMe4 in primary care is practical [32,34].

The study was conducted at 3 pediatric primary care clinics around Orlando, Florida, an urban area where the population at the time of the study was 57% White, 24% African American, and 33% Hispanic, with 16% living in poverty [35]; 2 of the clinics were part of a large health system, while 1 was privately owned. At the time of the study, the clinics treated between 212 and 622 patients aged 11-12 years, with 30%-90% of patients eligible for Medicaid and HPV vaccine initiation rates ranging from 8% to 44%. Each clinic participated in the federal Vaccines for Children program. All 3 clinics automatically updated the state immunization registry through a real-time linkage with their electronic health record systems.

## Methods

### Study Overview

Between March 2020 and March 2021, we evaluated the usability of ProtectMe4 using a think-aloud assessment and surveys [32]. Testing was paused from mid-March to mid-September 2020 due to disruptions in primary care caused by the COVID-19 pandemic. The think-aloud method involves direct observation of users interacting with the system, where participants are instructed to provide a “running commentary” of their thoughts. The observer encourages participants to continue sharing their thoughts through planned prompts [32]. The purpose of this evaluation is to address design features that participants identify as confusing or difficult. We conducted the think-aloud assessment in the intended setting to closely simulate the clinic workflow and provide users with a near-real-life experience of the app. After using the app, both parent and clinician participants were invited to complete a usability survey.

### Ethics Approval

The study was approved by the University of Florida Institutional Review Board (approval number 201602385).

### Participants

Clinicians and parents of 11- to 12-year-olds attending their child’s clinic visit were invited to participate. This age group was chosen because the United States Advisory Committee on Immunization Practices recommends universal HPV vaccination for 11- and 12-year-olds [36]. At each of the 3 clinics, clinicians who provided primary care to 11- or 12-year-old patients were invited to participate and complete an informed consent process. We aimed to recruit 3-4 parents of 11- to 12-year-olds at each clinic during their child’s visit. For clinics with multiple clinicians, we aimed to balance recruitment efforts among them. On days when 2 or more 11- to 12-year-olds had scheduled visits, research staff visited the clinics, and clinic staff invited parents of these children to participate in the study. Both parents and clinicians received US $25 for their participation.

### Methods for Data Acquisition

Our data acquisition process approximated our planned workflow. Our plan is for clinic staff to invite parents to use the app during check-in or triage [27]. When parents use the app, clinic staff will notify the clinician before seeing the patient, allowing the clinician to review the app’s responses either before or during the patient visit. However, for the think-aloud assessment, to minimize the burden on clinic staff related to participant consent, clinic staff invited parents using a brief script and provided an informational flyer explaining the study’s purpose during check-in or triage. When parents expressed interest, clinic staff alerted the research staff, and the research staff conducted informed consent in a private clinic room and carried out the think-aloud assessment and survey. After the parent completed these steps, the study staff aimed to show the app to the clinician before the clinician saw the participating parent and child.

After obtaining informed consent from parents and assent from adolescents, parents were provided with the ProtectMe4 app on an iPad (Apple Inc.). For security, the iPad had limited functionality, including restricted access to relevant websites, internet access confined to preset mobile Wi-Fi devices, and remote management. We used QuickTime (Apple Inc.) on a MacBook (Apple Inc.) to record the iPad screen and room audio, connecting the iPad and MacBook with a lightning cable. To minimize the training burden on clinic staff, research staff assumed the role of clinic staff by logging into the app’s parent interface and entering the child’s information to create a unique 1-time use identifier. Parents were instructed to use the tool as if they were on their own and to verbalize any thoughts they had. Research staff answered questions about the tool and prompted parents with guided questions (eg, “What do you think happens if you press that button?” or “What is being communicated on this page?”). Interviewers followed a semistructured guide to help probe parents about their experience with the app (see Multimedia Appendix 1).

After a parent used the app, research staff aimed to have clinicians use ProtectMe4 before seeing the patient. Clinicians were provided with the app and asked to use it as they would on their own, vocalizing any comments or issues they encountered. As a result of the clinicians’ limited time, research staff took notes on their responses rather than creating recordings.

After using the app, parents were immediately given a paper-based survey, while clinicians were asked to complete their survey by the end of the clinic day. The survey questions were adopted or adapted from the System Usability Scale (SUS), the Technology Acceptance Model, and related constructs of behavioral intention and technology self-efficacy [37-42]. The research team selected the most relevant constructs from these surveys to assess parents’ and clinicians’ perceptions of the app’s clarity, usefulness, ease of use, navigation, and presentation. The SUS was included only for clinicians, as future use was less relevant for parents.

### Methods for Data Analysis

To identify usability issues for parents, we reviewed the linked audio and screen recordings of their think-aloud activities. This helped us understand specific challenges with using ProtectMe4, including reasons behind their selections and their thought processes as they navigated the app. Three reviewers analyzed the recordings and documented their observations, which were then compiled into screen-by-screen reports detailing the comments parents made about the app. For clinicians, we reviewed staff notes from the think-aloud sessions. For both parents and clinicians, we compiled a list of instances where users had questions or encountered difficulties with the app. For each issue, the research team evaluated its significance and discussed potential adjustments. We report and address concerns that either (1) were common among users, or (2) had straightforward solutions that did not impact other aspects of the app. The time spent using the app was tracked using the app’s time log. For the survey, we calculated the frequencies of responses from both clinicians and parents. For clinicians, we calculated the SUS score on a 0-100 scale [42]. Each of the 10 items was scored from 0 to 4 based on Likert scale responses, with “strongly agree” assigned a score of 4. We summed the scores for all items and multiplied the total by 2.5 to obtain the final SUS score.

### ProtectMe4 App Description

**Figure 1:**
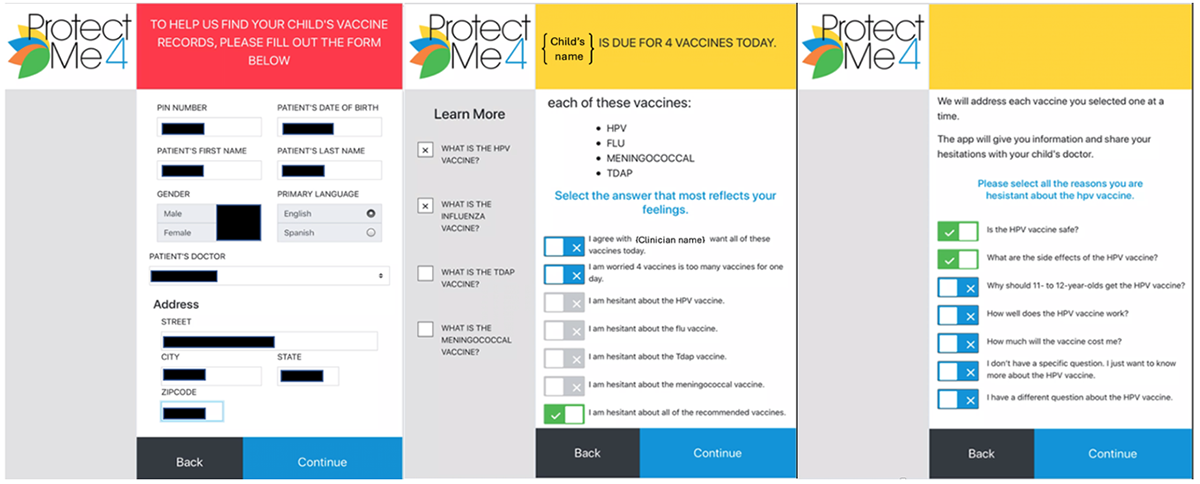
Parent ProtectMe4 interface with vaccine identification, vaccine display, and hesitations.

For parents, ProtectMe4 starts with an introduction screen that emphasizes the tool’s role in enhancing, rather than replacing, the conversation with their doctor. Parents then enter their child’s information, and the app submits a query to the state immunization registry to retrieve the recommended adolescent vaccines (Figure 1). The app presents a screen indicating which specific adolescent vaccines, those not previously received, are being recommended by the child’s clinician. Parents can then select which vaccines they want or are hesitant about. If a parent accepts all vaccines without hesitation or if the child is up-to-date on all vaccines, the app is complete. For each vaccine about which parents express hesitation, the app presents common hesitations derived from the literature. Parents select the hesitation(s) that best reflect their feelings. For each selected hesitation, the app provides tailored educational information addressing that specific issue. After reviewing the tailored informational screens, parents are prompted to indicate their interest in receiving the recommended vaccines.

**Figure 2:**
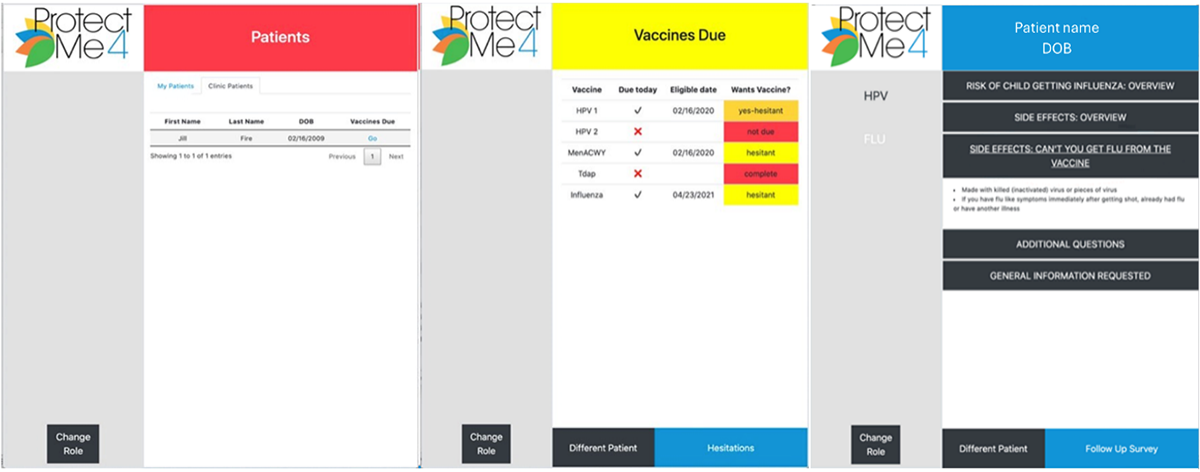
Clinician ProtectMe4 interface with patients, vaccine display, and hesitations.

For clinicians, the app begins with a list of patients whose parents completed the app that day (Figure 2). Clinicians can also access a tab labeled “clinic patients” to view other patients at their clinic. By selecting a child’s name, a table displays the adolescent vaccines, the child’s vaccine due dates, and the parent’s interest in each vaccine. For vaccines about which parents expressed hesitation, clinicians can view the specific hesitations and receive tips for addressing them. Finally, clinicians are prompted to record their vaccination intentions, including whether they plan to administer the vaccine that day, discuss the due vaccines, use the provided tips in their discussion with the parent, or schedule appointments for the second HPV vaccine dose.

## Results

### Parents

#### Workflow and App Timing

**Figure 3:**
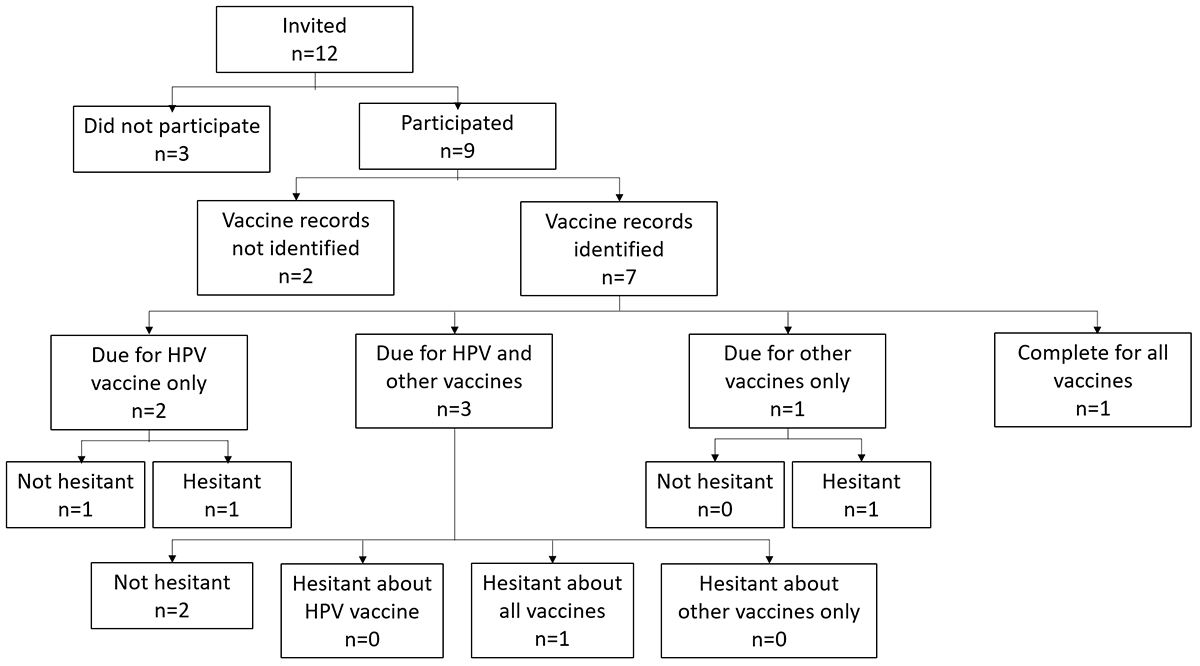
Flow of parents invited to participate. (Other vaccines include MenACWY, Tdap, and influenza. MenACWY: meningococcal conjugate vaccine against serogroups A, C, W, and Y; Tdap: tetanus, diphtheria, and pertussis.).

Among the 12 parents invited to use the app during their child’s visit, 9 (75%) participated (Figure 3). One parent (8%) declined because she was uninterested in vaccines. As a result of clinic staff forgetting to ask parents about the study at check-in, 2 parents (17%) were invited after meeting with the clinician; both declined, citing other time commitments.

Among the 9 participating parents, the app successfully identified and displayed the child’s vaccination records for 7 parents (78%). For these parents, the median time spent on the app was 9 (range 6-28) minutes. The average time spent on each section of the app was as follows: entering the child’s information (average 3 minutes, range 1 minute 30 seconds to 4 minutes), reviewing the consent (average 3 minutes 45 seconds, range 1 minute 43 seconds to 5 minutes 18 seconds), and exploring the vaccine educational material (average 3 minutes 11 seconds, range 0 minutes to 19 minutes 29 seconds).

#### Usability Themes

##### Overview

Think-aloud assessment results for parents were grouped into 2 main themes: (1) troubleshooting vaccine record identification and (2) clarifying the app content and purpose. Within each theme, we identified solutions for the issues encountered (Table 1).

**Table 1:** Think-aloud assessment–identified issues and corresponding adjustments.

Theme and identified issue		Adjustments	User group
**Troubleshooting vaccine record identification**			
	Records not found from parent-entered information	Improved instructions to read “enter address for where parent lived when your child last received a vaccine.” Additional instructions if the error is still present: “enter address from when your child was 6 years old.”	Parent
	Parent does not agree with the result of the displayed vaccination record	Response option added to the PM4 interface for parents to indicate “I do not agree with a displayed vaccine record.” This alerts the clinician to explore vaccination records further on the clinician dashboard.	Parent
**Clarifying ProtectMe4 app content**			
	Confusion about the inclusion of meningococcal vaccine	Language clarification made to specify that the recommendation is for the meningococcal conjugate vaccine against serogroups A, C, W, and Y and not the meningococcal B vaccine.	Parent
	Confusion about the process of selecting vaccine hesitations	Change instructional language in the app to improve clarity: “Select the questions that you have about the HPV vaccine.” Change the interface design: remove the “green checkmark” indicator when hesitation is selected. Replace with a radio button.	Parent
	Remaining discomfort about vaccine administration	Added an option at the end of parent interaction to express “I still have questions for doctor.”	Parent
	Increase neutrality of language in the app	Action buttons are renamed to “view” for patient records.	Clinician
	Excessive variation in the use of symbols such as “x” versus “checks”	Symbol use has been simplified and training materials developed to clearly articulate the purpose of symbols when orienting clinicians to the system.	Clinician

##### Troubleshooting Vaccine Record Identification

One of the 7 parents for whom the app successfully identified their child’s vaccine records initially failed to retrieve the records when entering their current address. The child’s vaccine records were successfully found when the parent entered their address when the child was 6 years old (likely the last time the child received a vaccine, if not receiving the annual influenza shot) [19]. Based on this finding, we will add instructions to the address fields advising parents to enter the address where they lived when their child last received a vaccine, with a prompt indicating that this might be when the child was 6 years old. Additionally, if the app does not find the child’s records, we will prompt parents to enter a second address, with repeated instructions suggesting that this could be a previous address.

Using a previous address did not allow the app to identify the child’s vaccine records for 2 parents. For both children whose records could not be identified, the clinic served a population with 90% Medicaid patients. Upon visual inspection of the children’s Florida SHOTS records by clinic staff, it was found that there were 2 records in the registry with different addresses for each of these children.

For the 7 children whose vaccine records the app successfully identified and displayed, the vaccination status was as follows: 2 children were due for HPV only, 3 were due for HPV and other vaccines (MenACWY, Tdap, or influenza), 1 was due for only other vaccines (MenACWY and Tdap), and 1 was up to date with all vaccines. In the case where the app indicated that the child was up to date on all vaccines, the parent reported that the purpose of the child’s visit on that day was to receive a vaccine. Upon inspection, it became clear that clinic staff had marked the vaccine as administered in the registry shortly before the parent used the app. This situation highlighted an additional use case not originally planned for in the system design: when a parent disagrees with the displayed vaccine record. To address this, we will add an option for parents to indicate disagreement with a displayed vaccine record, which will alert the clinician to review the vaccine record.

##### Clarifying the App Content

Among the 6 parents for whom the app displayed due vaccines, 3 agreed to have all recommended vaccines administered during their appointment, while 3 expressed hesitation about all the recommended vaccines (1 parent hesitated about HPV only; 1 about MenACWY and Tdap; and 1 about HPV, MenACWY, Tdap, and influenza). Additionally, 1 parent was confused about the inclusion of the meningococcal vaccine, mistakenly thinking it was only for students entering college. To address this confusion, we will clarify that the app is referring to the MenACWY vaccine, not the meningococcal B vaccine (MenB) vaccine.

When asked to choose between types of vaccine hesitations, the 2 parents who were hesitant about the HPV vaccine selected concerns about (1) safety and (2) side effects. Additionally, 2 of the 3 parents who saw the hesitation screens expressed confusion, believing that selecting a hesitation statement meant they were agreeing with the statement as true. For example, one parent said:

To me [the checkmark] indicates yes, instead of this is my question ‘cause I really don’t know if it is safe. Cause if I check it, I’m thinking that it means “yes, it is safe”.

To clarify that the app is assessing the topics the parent is concerned about, we will make 2 changes. First, we will change the text from “Please select all the reasons you are hesitant about the HPV vaccine” to “Select the questions you have about the HPV vaccine.” Second, we will replace the green checkmark used for selection with radio buttons.

To capture any direct effects of participating in the app, it concludes by asking parents, “Now that you have learned more about vaccines, which do you intend to get today?” One parent felt uncomfortable with this screen because she was still undecided and wanted to discuss it with her child’s doctor. To address this concern, we will add an additional response option: “I still have questions for the doctor.”

#### Parent Perceptions About App

Nearly all (8/9) parents completed the survey and reported that the app was acceptable, provided appropriate content, and was easy to use (Table 2). Most parents (7/8) indicated that ProtectMe4 made it easier to discuss vaccines with their child’s doctor and would recommend it to other parents. Three-quarters of parents felt that ProtectMe4 facilitated their decision-making regarding recommended vaccines. Although only about half of the parents (5/8, 63%) considered themselves skilled with technology, all reported that the app was easy to use.

**Table 2:** Percentage of users agreeing or strongly agreeing with the usability of ProtectMe4.

Source and usability item			Parent (n=8), n (%)		Clinician (n=4), n (%)
**Usability and user experience measure [37]**					
	The information in ProtectMe4 was clear^a^	8 (100)		4 (100)	
	ProtectMe4 provided enough information^a^	7 (88)		4 (100)	
	The information provided in ProtectMe4 had enough detail^a^	7 (88)		4 (100)	
	ProtectMe4 provided up-to-date information^a^	—^b^		4 (100)	
	ProtectMe4 gave me the information I needed in time^a^	8 (100)		4 (100)	
**TAM^c^ 1 [39]**					
	ProtectMe4 was useful	7 (88)		4 (100)	
	ProtectMe4 made it easier to talk to parents/my child’s doctor about vaccines	7 (88)		4 (100)	
	ProtectMe4 will enhance my effectiveness in providing vaccines	—		4 (100)	
	ProtectMe4 will be useful in vaccinating patients/deciding about recommended vaccines	6 (75)		4 (100)	
	Using ProtectMe4 increased my productivity	—		4 (100)	
	I recommend other parents use ProtectMe4	7 (88)		—	
	I predict I will use ProtectMe4	—		4 (100)	
**TAM 3 [40] and SUS^d^ [42]**					
	ProtectMe4 was easy to use	8 (100)		4 (100)	
**TAM 3 [40]**					
	I found it easy to get ProtectMe4 to do what I wanted it to do	8 (100)		4 (100)	
**Acceptance and usability measures [41]**					
	It did not take too many steps to find the information I needed in ProtectMe4	8 (100)		4 (100)	
	I liked the way the information in ProtectMe4 was arranged	7 (88)		4 (100)	
	I liked the color and layout of the pages in ProtectMe4	8 (100)		4 (100)	
	ProtectMe4 did not use confusing acronyms or abbreviations/words	7 (88)		4 (100)	
**Acceptance and usability measures [41] and TAM 1 [39]**					
	Learning something new with technology is easy for me	8 (100)		3 (75)	
	I consider myself to be a very skilled user of technology	5 (63)		2 (50)	
**SUS [42]**					
	I think I would like to use ProtectMe4 frequently	—		4 (100)	
	I found ProtectMe4 unnecessarily complex	—		2 (50)	
	I think I would need the support of a technical person to be able to use ProtectMe4	—		0 (0)	
	I think the various functions of ProtectMe4 were well integrated	—		3 (75)	
	I thought there was too much inconsistency in ProtectMe4	—		0 (0)	
	I would imagine that most people would learn to use ProtectMe4 quickly	—		4 (100)	
	I found ProtectMe4 very cumbersome to use	—		0 (0)	
	I felt very confident in using ProtectMe4	—		3 (75)	
	I needed to learn a lot of things before I could get going with ProtectMe4	—		0 (0)	

^a^% item scale from almost never to almost always. Percentages are reported for most of the time or more.

^b^Not asked.

^c^TAM: Technology Acceptance Model.

^d^SUS: System Usability Scale.

### Clinicians

#### Clinician Participation

All clinicians who provided primary care to 11- or 12-year-olds agreed to participate (7/7). Of the 7 consented clinicians, 4 had patients who used the app as part of their regular clinical care and were subsequently invited to take part in the usability assessment.

#### Workflow and App Timing

Consistent with the planned workflow, the clinician viewed the app before seeing the patient in 4 out of 9 instances (44%). However, despite the study staff’s best efforts, the clinician viewed the app after seeing the child in 2 instances (22%). In 1 case, the clinician had already ordered all due vaccines before reviewing the app. In the other case, the child was at the clinic for an acute visit and was due for HPV, MenACWY, and Tdap. The parent had expressed interest in receiving all vaccines. However, after the clinician viewed the app, she returned to discuss with the family and explained that she would not recommend the vaccines during this visit due to the child having a mild infection. For 3 patient participants (33%), the study staff did not prompt the clinician to review the app’s responses because either the parent disagreed with the app results (n=1) or the app did not identify any vaccines for the child (n=2).

The median time that the 4 participating clinicians spent on the app per patient was 95 seconds (range 5-240 seconds). One clinician spent only 5 seconds on the app, as they merely reviewed the results without completing the questions. Another clinician, who used the app for 240 seconds, experienced difficulties with the app accepting her log-in information.

#### Usability Themes

##### Think-Aloud Assessment Results

Think-aloud assessment results for clinicians were categorized into 2 themes: (1) trust in the app’s vaccine results and (2) clarification of the app content.

##### Trust of App Vaccine Results

While the app is intended to supplement the clinician’s review of the electronic health record for vaccinations, observations revealed that clinicians were not consistently relying on the app for vaccine recommendations. One clinician explicitly stated she would “double check her system to see what the patient is due for.” We did not modify the app content based on this issue as it pertains to clinician behavior rather than app functionality.

##### Clarifying the App Content

Clinicians were able to navigate the app successfully; 3 out of 4 clinicians found the app easy to follow, quickly locating the patient they were searching for. They also demonstrated their ability to find other patients and found the hesitations and tips sections easy to understand and use. One clinician expressed the ease of use by stating: “I am not a techie and think the app was very easy to use and straightforward.” All clinicians selected “Go” to view the due vaccines for their patients. One clinician stated that “Go” could be changed to something more neutral. Thus, we will change this link to say “View.” All 4 clinicians completed the in-app survey about intentions to vaccinate.

One clinician commented that there was “too much going on with X’s and checkmarks.” While only mentioned by one clinician, we will address this concern because it is like a parent concern. Thus, we will streamline the use of symbols and develop training materials and brief educational sessions to help clinicians navigate the app more effectively.

#### Clinician Perceptions About App

All 4 clinicians were unanimously positive about the acceptability, appropriateness, and feasibility of using ProtectMe4 (Table 2). The mean SUS score was 87.5 (SE 2.5). Although only 2 out 4 (50%) clinicians considered themselves skilled technology users, all predicted they would use ProtectMe4 if it were available.

## Discussion

### Answer to Study Objectives

This mixed methods evaluation demonstrated that ProtectMe4 was both usable and acceptable to parents and clinicians in real-world pediatric primary care settings. While the app’s compatibility with workflow was generally positive in terms of user time, challenges remained in clinic staff offering the app to parents and ensuring clinicians viewed it before seeing patients. The think-aloud assessments showed that the app was mostly clear to users and highlighted areas for refinement and limitations. Finally, both parent and clinician responses to the usability survey were overwhelmingly positive, indicating that the app is acceptable, appropriate, and feasible. Although further workflow revisions may be necessary, ProtectMe4 has the potential to enhance the efficiency and effectiveness of vaccine conversations between parents and clinicians.

### Workflow

On a positive note, most parents were able to complete the task in the app during the observed waiting times in pediatric clinics, with completion times ranging from 22 to 38 minutes [27,43]. Additionally, clinicians required minimal time to extract the necessary information from the app. However, improvements in ProtectMe4’s compatibility with clinic workflow, an important aspect of the Consolidated Framework for Implementation Science [24], are likely needed before full implementation. First, some usability issues could be mitigated by increasing staff members’ and clinicians’ understanding of the underlying principles and rationale for adopting the ProtectMe4 app. To prevent parents from viewing incorrect information, staff should ensure that vaccines are recorded in the immunization registry after parents have used the app. Additionally, to avoid conflicting information between the app and health care providers’ recommendations, clinicians should align the app’s use with the clinic’s practices regarding vaccine administration during acute visits. Second, the proposed workflow involving 2 staff members—the front office staff and the triage nurse—responsible for providing the iPad to parents is likely necessary. This 2-staff-member system addresses the issue where the single-staff-member approach used in the usability assessment missed several parents [27]. Third, the ProtectMe4 app workflow will require improved coordination among clinic staff to ensure that clinicians have the opportunity to view the app before seeing the patient.

### Usability of App Content

Three issues identified in the ProtectMe4 app are likely relevant to other apps designed to assist parents in considering vaccines for their children. First, parents highlighted areas where the wording could be clarified or offer greater flexibility. Improving clarity and flexibility is essential to enhancing parent autonomy and trust, which are critical for effective communication. For example, clarifying the intent behind selecting hesitations and providing an additional option to indicate the desire to continue the conversation about vaccination intentions could be beneficial [44,45]. Second, many 11- to 12-year-olds may not have received vaccines since the age of 6 years, leading to outdated addresses in immunization registries. In addition to vaccine look-up, as was the case for ProtectMe4, this issue can limit the effectiveness of registry-based vaccine reminders for this age group [46,47]. Third, record duplication in the Florida vaccine registry—common in other vaccine registries—hindered the app’s ability to accurately identify vaccines for highly transient families [48]. Deduplication of multiple registry entries requires deterministic or probabilistic matching processes and human review [48,49]. In Florida, immunization records can only be combined by contacting the Department of Health, which then reviews and deduplicates the records [50]. As a result of this complexity, duplicate records are often considered together for clinical care but are not deduplicated in the registry itself.

### Clinicians’ and Parents’ Perspectives

Compared with other apps and proposed usability benchmarks [37,39,40,42], ProtectMe4 achieved high scores in user satisfaction, perceived usefulness, and acceptance, with agreement rates ranging from 75% to 100% for parents and 100% for clinicians. This indicates that ProtectMe4 is a promising innovation for primary care implementation. Additionally, the clinicians’ SUS score of 87.5, equivalent to a grade of A+, surpasses the SUS score for Amazon, which is 81.8 [51].

### Limitations and Strengths of the Study

The study had 3 important limitations. First, only 9 parents and 4 clinicians participated. Although these numbers are small, research indicates that 5-9 participants can identify up to 80% of usability issues, with diminishing returns for additional participants [52]. Second, the study visits occurred just before and during the COVID-19 pandemic, before the availability of the COVID-19 vaccine. The pandemic may have influenced our results in 2 ways: parents willing to participate in the study may differ from the general population, as they attended in-person visits during a time when many others may have avoided them. However, while acute visits remained low throughout the study period, well visits for this age range were only notably affected between mid-March and August 2020, when app testing was paused [53,54]. Additionally, the COVID-19 pandemic may have influenced parents’ views on the app; however, predicting the direction of this influence is challenging due to the varied shifts in vaccine opinions during the pandemic [55]. Third, while our focus was on the age group for which universal vaccination is recommended in the United States, the vaccines are also recommended for unvaccinated adolescents between 13 and 17 years of age, and there have been growing calls for HPV vaccination starting at ages 9-10 years [19,56]. It is unclear whether parents of children in these different age groups might encounter different usability issues with the app.

The study also had 3 notable strengths. First, we gathered both qualitative and quantitative data on ProtectMe4’s usability, providing a more comprehensive understanding than either method alone [57]. Second, think-aloud testing was conducted during real clinical visits, enabling the simultaneous identification of app content and workflow issues. Third, given the increased importance of tools to facilitate vaccine discussions in light of COVID-19 vaccine availability and challenges to vaccine confidence, this study’s findings are particularly timely and relevant [58].

### Conclusions

Innovations that enhance parent-clinician conversations about vaccination are crucial, as clinicians are the most trusted source of vaccine information. Despite this, they often feel uncomfortable and lack the time for in-depth discussions with hesitant parents [17,18]. ProtectMe4 demonstrated usability and acceptability for both parents and clinicians in real-world clinical settings. The identified usability improvements are expected to enhance the app’s implementation and effectiveness, particularly in promoting adolescent vaccinations, including the HPV vaccine. Allowing parents to express their concerns through an app while waiting to see their child’s physician may lead to more focused and meaningful conversations during primary care visits. Further evaluation of the ProtectMe4 app may demonstrate its effectiveness in increasing HPV vaccination rates among adolescents.

## Appendix

Multimedia Appendix 1 Semistructured guide for parent think-aloud.

## Abbreviations

HPV: human papillomavirus
MenACWY: meningococcal conjugate vaccine against serogroups A, C, W, and Y
MenB: meningococcal B vaccine
SUS: System Usability Scale
TAM: Technology Acceptance Model
Tdap: tetanus, diphtheria, and pertussis
