# Supplementary material for: Usability of a Web-Based App for Increasing Adolescent Vaccination in Primary Care Settings: Think-Aloud and Survey Assessment
Source: JMIR Form Res. 2024 Sep 19;8:e56559. doi: 10.2196/56559 (PMC11450356; doi:10.2196/56559)
Supplement: Multimedia Appendix 1 [file formative_v8i1e56559_app1.docx]

**Appendix 1. Semi-structured Guide for Parent Think-Aloud**

Interviewers were provided the following questions to help prompt parents to express their thoughts during the use of the *ProtectMe4* app.

**Clinician Overview Screen:**

- In your own words, what is this screen telling you?
- What do you think you could click on this screen and what would happen?

**Patient Information Screen:**

- As you complete this screen, tell me if you find any fields difficult or confusing?

**Patient Confirmation**

- What do you think the purpose of this screen is?

**Consent**

- What do you think the purpose of this screen is?
- How easy is it to understand the content of the informed consent?
- How easy is it to navigate through the consent and sign it?

**Addendum**

- What do you think the purpose of this screen is?

**Close survey screen**

- What do you think you should do here?

**IF PATIENT IS UP TO DATE**

- What do you think you should do at this point?
- How do you log out?
- What should you do with the iPad?

**IF VACCINES DUE**

- How do you feel about interacting with this page?
- Which vaccines is your child due for?
- What can you click on within this page?
  - What happens when you click the “learn more” buttons?

**First vaccine hesitation selection page (any vaccine)**

- What is the point of this screen?
- How would you enter your own question in (if you had one)?
- What do you expect to happen when you hit continue?

**First viewed hesitation page (any vaccine)**

- What do you think you can click on within this page?
- What do you think about how the font/readability of this screen?
- What do you think happens when you click on the side buttons?
  1. Information buttons?
  2. Skip section button?
  3. Our story button?
  4. Skip to end button?
- At this point, how would you exit the application if you wanted to do so?
- Where do you think the back and continue buttons take you?

**Second viewed hesitation page**

- Where do you think the back/continue buttons take you now?

**Second hesitation select screen (if applicable)**

- What do you think when you land on this page?
- What is the point of this page?
- What will happen when you hit continue?

**Hesitation screen**

- At this point, how would you exit the application if you wanted to do so?

**Prompts for subsequent hesitation select/information screens**

- At this point, I’m going to let you use the application a bit without asking too many questions, please use the application as you normally would and talk about your impressions as you use it

**Follow-up screen**

- What do you think the point of this screen is?
- What do you think about this screen?
- What would you do if you still had hesitations about a vaccine?

**More information screen**

- What is the reason for this screen?
- Thoughts about using the screen?

**Logout**

- What would you do at this point of the process?
